# A Review of PFAS Destruction Technologies

**DOI:** 10.3390/ijerph192416397

**Published:** 2022-12-07

**Authors:** Jay N. Meegoda, Bruno Bezerra de Souza, Melissa Monteiro Casarini, Jitendra A. Kewalramani

**Affiliations:** Department of Civil and Environmental Engineering, New Jersey Institute of Technology, Newark, NJ 07102, USA

**Keywords:** per- and polyfluoroalkyl substances, destruction technologies, separation technologies, per- and polyfluoroalkyl generated from ion exchange resin

## Abstract

Per- and polyfluoroalkyl substances (PFASs) are a family of highly toxic emerging contaminants that have caught the attention of both the public and private sectors due to their adverse health impacts on society. The scientific community has been laboriously working on two fronts: (1) adapting already existing and effective technologies in destroying organic contaminants for PFAS remediation and (2) developing new technologies to remediate PFAS. A common characteristic in both areas is the separation/removal of PFASs from other contaminants or media, followed by destruction. The widely adopted separation technologies can remove PFASs from being in contact with humans; however, they remain in the environment and continue to pose health risks. On the other hand, the destructive technologies discussed here can effectively destroy PFAS compounds and fully address society’s urgent need to remediate this harmful family of chemical compounds. This review reports and compare widely accepted as well as emerging PFAS destruction technologies. Some of the technologies presented in this review are still under development at the lab scale, while others have already been tested in the field.

## 1. Introduction

Per- and polyfluoroalkyl substances (PFASs) have been designated as emerging contaminants of concern since the early 2000s [1,2,3,4]. PFAS compounds have been detected in surface and groundwater at more than 2000 locations in the US, with the highest concentrations at former Department of Defense (DOD) fire-training facilities [2,3,5,6,7]. PFASs comprise a diverse group of synthetic chemicals used for over 90 years [8]. It is a complex group of chemicals, which consists of compounds with carbon-fluorine solid bonds (C-F) and is the shortest and strongest known covalent bond in nature and is responsible for the thermal and chemical stability of PFASs [9,10,11,12,13]. In conjunction with their ability to act as surfactants, these properties make PFASs ideal for a wide range of industrial and commercial applications [1].

**Figure 1:**
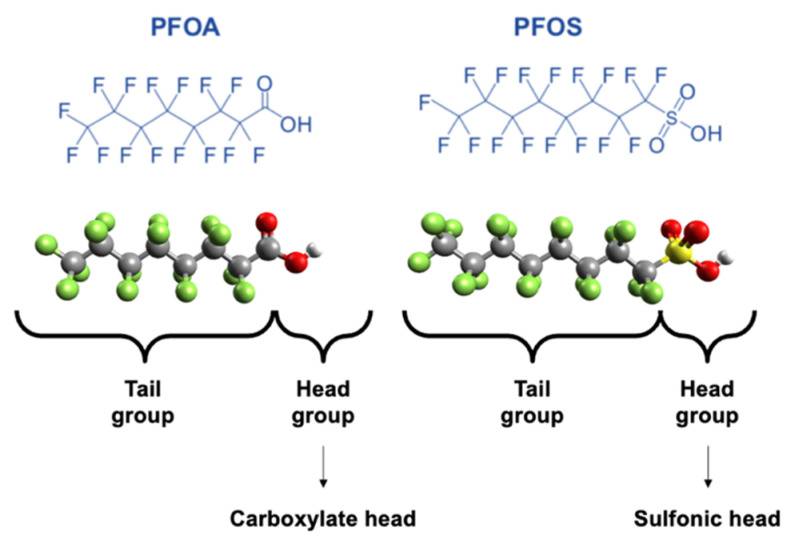
PFOA and PFOS chemical structures.

Due to the low surface tension and wetting properties of PFAS, they are used in paint additives, non-stick cookware, and firefighting foams [9,14,15]. Unfortunately, these same properties also rendered PFASs bio-accumulative, toxic to both the environment and human health, and ubiquitous in the environment [16,17]. Perfluorooctane sulfonic acid (PFOS) and perfluorooctanoic acid (PFOA) have been the most extensively manufactured and hence most frequently detected PFASs in the environment [18,19,20,21,22,23,24]. Figure 1 shows the chemical structure, the three-dimensional view, the tail group (C-F bonds), and the head groups of the carboxylates and sulfonics of these two molecules, PFOA and PFOS. Significant sources of PFAS released to the environment include fire training/fire response sites, industrial sites, landfills, and wastewater treatment plants/biosolids [25].

The most widely used non-polymer PFASs are perfluorooctanoic acid (PFOA), perfluorooctanoic sulfonic acid (PFOS), and perfluorohexane sulfonic acid (PFHxS) [26,27]. For the past five decades, mixtures of PFAS were added as film-formers and foam stabilizers in aqueous film-forming foams (AFFFs) and film-forming fluoroproteins (FFFPs). They have been used as fuel repellents to extinguish flammable aircraft fuel fires in the aeronautics industry [28,29,30]; however, PFAA precursors found in AFFF are also converted over time into PFASs and have lasted for decades in the environment [15,31]. PFASs have been detected in the local streams, soil, plants, and animal tissues throughout these sites. The presence of PFAS food packaging has been cited as an important pathway for human exposure other than direct consumption of PFAS-contaminated water. Studies in humans have discovered that PFASs can lead to cancer, problems in kidney function, metabolic disruption, and many other health issues.

The release of PFASs into the environment has become a growing concern of national and regional regulatory agencies due to the resistance of PFASs to natural degradation and their persistence in animals, humans, and the environment [27,32,33]. For this reason, use restrictions and other regulations for PFASs have been implemented around the world. The United States Environmental Protection Agency (USEPA) has developed advisory limits on PFASs in drinking water since 2009. The most recent guideline implemented in June 2022 has advisory limits of 0.004 parts per trillion (ppt) of PFOA, 0.02 ppt of PFOS, 10 ppt of GenX chemicals, and 2000 ppt of PFBS. In May 2022, the USEPA added five more PFAS compounds for site cleanups (perfluoronanoic acid (PFNA), PFHxS, perfluorononanoate, perfluorooctanoate, and perfluorohexanesulfonate) based on risk-based values for regional screening levels (RSLs).

Conventional water and wastewater treatment facilities are not capable of removing PFASs [15]. The PFAS effluent concentrations can be significantly higher when compared to influent levels due to PFAS-like precursor compounds breaking down within such treatment systems [30,34,35,36,37,38,39,40,41]. Therefore, numerous studies have developed technologies to capture these pollutants in drinking water sources. These technologies include ion exchange resin (IXR), granular activated carbon (GAC), nanofiltration (NF), and reverse osmosis (RO). Even though removal technologies have been proven effective in PFAS separation or adsorption, they do not eliminate or destroy PFASs. These are only interim actions involving the physical mass transfer (sequestration) of PFASs.

Separation technologies such as IXR and GAC can momentarily remove the PFAS from a specific medium; however, they remain in the environment and continues to cause health risks. GAC and IXR are currently the most common or widely accepted treatment options for PFAS removal from groundwater and drinking water. The IXR systems are more expensive than GAC, but IXR applications are gaining popularity over GAC due to the higher adsorption capacities of IXR, as well as shorter contact times and smaller equipment footprints. More importantly, IXR can be regenerated on-site to a nearly virgin capacity and hence can be used repeatedly, whereas the on-site regeneration of GAC is not feasible. The brine solution or fractions (still bottom-SB) resulting from IXR regeneration contains high concentrations of PFASs (typically in ranges of ppm), salt, and residual organic compounds. Worldwide, the high concentrations of PFASs generated from IXR technology are currently stored in secure sites until suitable destruction technology is identified. Hence the purpose of this publication is to facilitate the discussion on the selection of the best available destruction technology for the high concentrations of PFASs generated from IXR technology for the complete destruction of PFAS compounds.

Even though destructive technologies are still in the development stage, they have shown great promise to destroy PFAS compounds and provide a solution to effectively address society’s urgent need to remediate this harmful family of chemical compounds. Hence, this review aims to discuss and compare the most promising PFAS destructive technologies that are in the development stage. Some of the technologies presented in this review are still under development at the lab scale, while others have already been tested in the field.

The following technologies are discussed in this review:

- Electrochemical oxidation;
- Plasma;
- Photocatalysis;
- Sonolysis;
- Supercritical water oxidation;
- Thermal degradation/incineration.

Please note that despite extensive published experimental and numerical research on the degradation or mineralization of PFASs, there are still major gaps in the experimental data, which makes it challenging to perform a complete evaluation of the efficiency of those technologies. There are several unanswered questions to obtain a clear picture to allow the technology to be optimized. This review article aims to provide a comparative analysis to the scientific community on PFAS destruction technologies by comparing the widely studied methods as well as the most promising emerging methods. Then, it provides the benefits and drawbacks of those technologies.

There is an urgent societal need to investigate cost-effective and safe PFAS mineralization technologies with minimal adverse environmental impacts. Hence this topic is extremely relevant as it will help the user to understand the mechanics of PFAS destruction technologies at the development stage and will allow optimization of those to have a cleaner environment free of PFAS contaminants.

## 2. Electrochemical Oxidation

**Figure 2:**
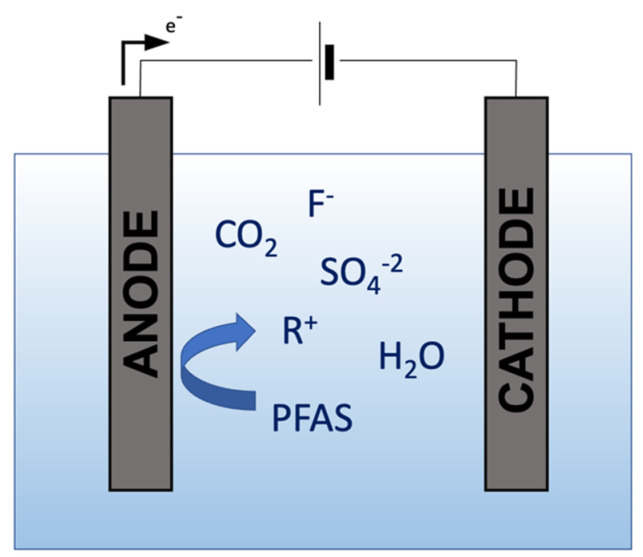
Mechanism of Electrochemical Oxidation.

The electrochemical oxidation (EO) method oxidizes and reduces organic pollutants by applying an electrical current through a conductive solution between an anode and a cathode. The contaminants are either adsorbed and degraded at the electrode or in the liquid medium [15,42]. The EO method has been shown to break PFASs into environmentally benign products through direct and indirect reactions. The direct oxidation results from electron transfer from the PFAS compound to the anode. At the same time, indirect mechanisms involve the creation of powerful oxidants known as radicals electrochemically [43,44,45]. A series of reactions separate intermediate products from the parent compound and are subsequently defluorinated [46,47,48]. The treatment of long-chain PFAS has demonstrated removals as high as >99%. On the other hand, short-chain PFASs are generally more challenging for the EO method to degrade [30], and it can even increase the PFAS concentration after treatment due to the conversion of precursors [44,49,50]. The EO mechanism used for PFAS destruction can be observed in Figure 2.

The time of treatment of PFAS via EO depends on a wide range of variables such as electrode characteristics and surface area, initial PFAS concentration (presence of co-contaminants), efficiency target, and voltage. Recent studies have identified the use of reactive EO membranes can significantly expedite the reaction time [51]. Currently, most of the experiments are performed in laboratory-generated waste streams [45]. The real-world PFAS waste will likely need an extended treatment time or reduced performance and a decrease in the electrode lifetimes [52]. The reader is referred to Veciana [30], who compares recently published PFAS treatment with EO using real contaminated samples.

According to an evaluation in the literature [50], the kinetics rates of PFAS EO degradation were optimized by increasing the applied electrical current densities in the range of 20–350 A/m^2^. Evaluation of current density efficiency showed that 50 mA/cm^2^ produced better efficiency on PFAS degradation when compared to 10 and 20 mA/cm^2^, presenting removal of PFBA > 95% and perfluoroalkyl acid (PFAAs) 99% within 8 h of treatment of synthetic PFAS samples [49]. The treatment time and energy consumption can be significantly reduced when working with high concentrated PFAS streams. One method for increasing the concentration would be to use a coupled system containing the EO electrodes and a separation technique such as ion exchange IXR, nanofiltration (NF), or membrane filtration. These treatment trains have been proven to be extremely effective and, indeed, can potentially decrease energy consumption by more than 50% [30,52,53,54]. Specialized electrodes with tracer metals acting as catalysts have shown superior performance.

Advantages: EO is a superior oxidation technology in a few areas, such as working at low temperatures without adding harsh chemicals [15,42]. The strong PFAS oxidation performance, low-volume application, and relatively low environmental impact when related to other destruction technologies are some of the advantages of this technology [30,47]. Furthermore, hybrid destructions systems between EO and IXR and concentrated retentate from nanofiltration and reverse osmosis processes [44,50,54] have demonstrated high efficiency of PFAS destruction. EO effectively treats PFASs in synthetically prepared solutions and actual contaminated groundwater and wastewater with high destruction rates [30,48]. Additionally, it permanently destroys PFASs, does not require removing secondary waste, and does not require additional chemicals or pretreatment steps [15,55].

Disadvantages: Scaling up this technology is the main challenge for the full-scale application of EO for PFAS treatment. This is due to the low PFAS concentrations, high energy consumption, and expensive initial costs due to the high cost of the acquisition of electrodes [30,50]. The most commonly used electrode is boron-doped diamond (BDD); besides being challenging to produce, it costs approximately $7125/m^2^ [56]. Therefore, the study on PFAS degradation through EO is still limited to the laboratory scale [15]. New parameters such as electric field amplitude and the shape of the reaction chamber could significantly affect the degradation efficiency of PFASs [49,50,57,58].

Despite the excellent PFOA and PFOS degradation rates of 70–97.6%, as shown in Table 1 EO treatments of actual groundwater and wastewater with short-chain PFAS have either shown an increase in the concentrations or low removal rates [30]. This leads to the assumption that the breaking of C-F bonds of long-chain PFASs is susceptible to transformation products, including shorter-chain perfluorinated carboxylic acids (PFCAs) and fluoride [45,47,59,60]. Therefore, the sequential defluorination step may be required to fully mineralize PFAS molecules, resulting in the negative formation of short-chain compounds [61,62].

One alarming problem of the electrical chemical system for polluted water treatment is the products of its interaction, such as the formation of hydrogen fluoride vapor, which is highly corrosive [15,63]. The formation of oxidation byproducts such as perchlorate and chlorate has been reported in the literature after PFAS destruction with a BDD anode [43,45,49,64,65,66] and Ti/RuO_2_ anode [63], as well as the formation of bromine products [42,67,68]. Specifically, for water containing chloride, the formation of toxic chlorine (Cl_2_) was observed, although the mechanism is still unknown [42]. These harmful chemicals possibly originated from electrodes used as anodes in EO treatment, which mostly include toxic heavy metals that might be released into the treated water [42]. Hence, further studies to understand the formation of toxic bioproducts are necessary.

### Field Applications

Bench-scale: The transformation pathways for the chemical precursors of PFAAs present in AFFF during electrochemical treatment of two naturally impacted groundwater was studied for a fire training area where AFFF was used and for unknown AFFF contamination (subsequently spiked with 3M AFFF) [45]. This research suggested that the precursors from the “aged contamination” found in the fire training sample were degraded without the generation of substantial amounts of PFAAs. While the “freshly” spiked groundwater, with a higher presence of PFOS, transformed to primarily PFCAs, with subsequent defluorination. The aim of this study was the analysis of the PFOA and PFOS degradation efficiency due to important parameters of the reactor configuration, where two different batch reactors were compared for a Magnéli phase Ti_4_O_7_; electrode. This included applying the same current density (10 mA/cm^2^) to a simple one cathode and one anode setup and a double reaction area configuration, including two cathodes positioned on each side of the anode. This last format is broadly applied to the EO treatment of industrial wastewater [69]. Results from this study showed, for the same initial concentration, the two-sided reactor setup increased the efficacies of the EO on PFOA destruction to a half hour, resulting in a non-detectable level after 4 hours of treatment. Also, substantial treatment time reduction was observed for PFOS, and an explanation for this enhancement most likely would be the larger anode area of the doubled cathode setup.

Still, bottom: The interference from OM and co-contaminants on PFAS removal efficiency was identified by comparing the treatment of actual SB samples and synthetic spent regenerant solutions by applying the same current density (50 mA/cm^2^) on the BBD electrolysis system. The results suggested that the slower kinetics for PFAS removal for the real SB were due to the interference of OM and co-contaminants in the matrix. The TOC content of the mixture was reduced by 18.5% after 8 h of treatment of the actual SB solution compared to 67% for the synthetic solution [49].

Treatment train: The pioneer on-site pilot-scale treatment train of IXR with Ti_4_O_7_; electrodes EO system applied primary separation technology, removing and concentrating PFAS from groundwater into a small volume of SB waste, which was subsequently treated on-site by destructive EO technology. After 80 and 85 h in the reactor, 74.1–96.8% of PFAS precursors and 9.2–94.0% of four long-chain PFAAs (PFHxS, PFHpS, PFOA, and PFOS) were removed. Also, from 2.5 to 20.4% of short chains (i.e., PFBS, PFPeA, PFHxA, PFHpA) were also removed after 40 h of supplemental treatment in a bench-scale off-site study [44]. Compared with EO as a solo treatment, the effluent from IXR in the treatment train approach already contained rich salt concentrations in the spent resin, removed the need to add dosing salts, provided high ionic strength, and required much lower energy consumption for the degradation of PFASs.

## 3. Plasma

**Figure 3:**
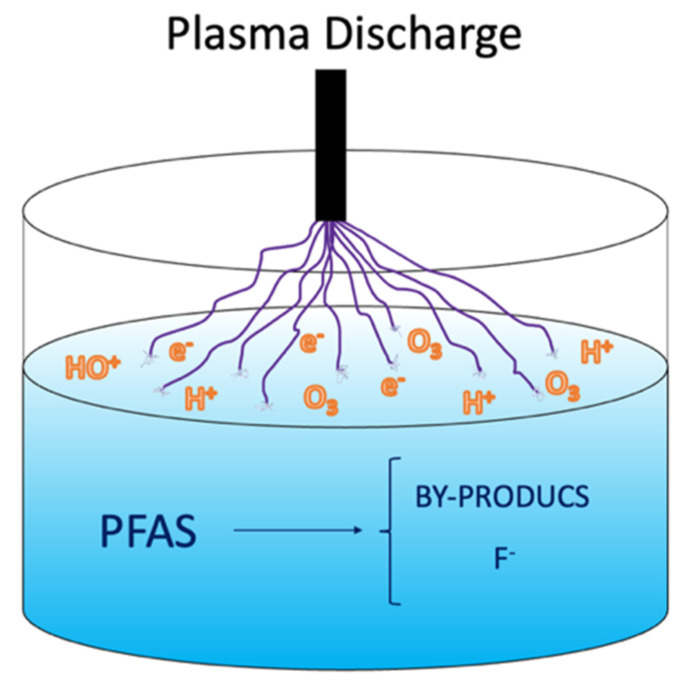
Non-thermal plasma approach for PFAS mineralization.

A plasma is an electrically charged gas created when adding energy which induces ionization of the gas molecules [27]. In plasma-based water treatment, highly reactive oxidative and reductive species are formed in response to the electrical discharge formed between two electrodes in the vicinity of liquid water [70,71,72]. The electrical discharge and its liquid interaction are shown in Figure 3, which conveys the mechanism behind the plasma technology. Additionally, temperature increases in the proximity of the discharge, the generation of shockwaves, and UV light emission occurs inside the reactor. Non-thermal plasma (NTP) is preferable for treating water contaminated with PFASs because NTP consumes a low level of energy at atmospheric pressure in air or with supporting gasses (He, Ne, Ar, O_2_ and N_2_) [73], showing higher excitation selectivity and energy efficiency than that for thermal plasma [15,74].

The NTP can be generated via diverse methods, including spark discharge, corona discharge, glow discharge, dielectric barrier discharge, and gliding arc discharge. The electrons are energized, and their temperature will be much greater than the gases in the environment. Consequently, the electrons will constantly collide with the gas’s atoms, generating electrons, radicals, ions, and photons [15,74]. The PFAS molecules are adsorbed onto the interface of the water bubbles, where the positively charged or negatively charged section of the PFAS collides with the ions with the highest energy in the plasma state [15,75,76]. Since the plasma discharge is a complete process, no additional chemicals are required to perform the treatment [73]. Argon bubbling has been reported to be the best-performing plasma reactor for treating surfactant-like compounds such as PFAAs [72,77,78]. The efficiency of plasma technology varies widely. It depends on several factors, for example, the reactor, electrode material, conductivity, applied voltage, PFAS type, pulse repetition rate, energy input, pH of the solution, liquid and gas temperature, liquid conductivity, and gas input [48].

Advantages: NTP is a promising technology for PFAA destruction [15]. PFAS destruction is more significant than other leading water treatment technologies [78,79]. The short treatment time compared to other PFAS degradations technologies is another advantage. Singh et al. showed that the presence of co-contaminants does not impact the effectiveness of plasma technology in treating PFAS [72]. Stratton et al. showed that the primary chemical reactivity occurs at the plasma-liquid interface. Hence, plasma-based water treatment appears to be less sensitive than most other treatment processes to the presence of organic and inorganic co-contaminants [78]. Moreover, Singh et al. noticed that the plasma treatment process appears to be a promising and efficient technology for removing PFAS from high-conductivity water [72,78]. NTP can effectively remove both short and long-chain PFAS [72], whereas short-chain PFAS compounds require longer treatment times than long-chain PFAS compounds.

Disadvantages: Some characteristics of contaminated water can directly reduce the treatment efficiency of NTP, such as pH, the concentration of OM, and the nitrate concentration. NTP technology has been used to mineralize PFAS with OM to obtain significant degradation rates. This suggests that the OM can play a competitive role in the reactive species generated by NTP with an overall decrease in the transformation rate of recalcitrant pollutants with the dissolved OM [73].

Previous studies about pH correlation with PFAS degradation show different effects. Some showed that acidic conditions resulted in more efficient reactions produced by oxidative species formation [15,74,80]. Additionally, the treatment of PFAS with plasma makes the treated water acidic; hence correction of the resulting low pH is necessary for subsequent use. Lastly, the way that the plasma method mineralizes PFAS is still not yet fully understood, and divergent pathway theories about its process are used in the literature. The formation of shorter-chain PFAS is commonly observed. The percentage conversion and the nature of the formed by-products depend on the experimental parameters [73]. Therefore, field demonstration experiments are limited to small-scale reactors.

### Field Applications

Pilot-scale: Singh et al. used a 4 L pilot-scale plasma reactor for PFAS removal from contaminated water from the United States Air Force Investigation Derived Waste (IDW). In this rector, argon was pumped through a submerged gas diffuser to form a layer of foam containing surfactant-like contaminants (e.g., PFAS) at the plasma−liquid interface [72]. PFOA and PFOS concentrations below the USEPA’s health advisory concentration (HAL) were achieved in 50 min. A study by Kazwini et al. indicated that the presence of co-contaminants does not impact the effectiveness of the plasma process in treating PFASs, showing below the detection limits of volatile organic compounds after treatment [27].

Treatment train: Aiming to optimize the treatment time and energy consumption of PFAS destruction using a plasma reactor and create a complete PFAS treatment solution, a modified technology to treat low conductivity groundwater by combining a train of IXR and a plasma reactor was presented in the literature [79]. Implementing two identical reactors in series to minimize the treatment time, the destruction of high concentrations was done in the first reactor and the refined residue at a low concentration in the second reactor. This method made it possible to remove long-chain PFAAs at an efficiency of >99% within 120 min and short-chain PFAAs of >99% after 6 hours of treatment. Observation of chlorite and chlorate concentrations after treatment showed significantly higher levels of chlorate concentrations of 6–720 mM, much higher than the USEPA’s HRL of 210 μg/L for drinking water. The energy consumed for the mineralization of PFAS compounds for this system with long-chain PFAAs, short-chain PFAAs, and precursors fluctuated between 1560 and 2370 kWh/m^3^ [79]. Additional data for this treatment can be found in Table 1.

## 4. Photocatalysis

Photocatalysis is a technology where a substance is activated due to the adsorption of a photon and in the presence of a photocatalyst which will accelerate the destruction reaction rate [81]. The photocatalyst substances are generally semiconductors. Recently, photocatalysts have been increasingly used because of their potential applications in solar energy conversion and environmental purification. Photocatalysis has enormous potential to treat organic contaminants in water and air [82]. The photocatalysis technology is an advanced oxidation process (AOP); consequently, it is applicable for the oxidation of a wide range of organic contaminants [82]. Heterogeneous photocatalysis substances have shown to be the most applicable due to their efficiency in degrading recalcitrant organic compounds, with similar characteristics as PFAS [82]. In past years, several studies have used heterogeneous photocatalysis oxidation to degrade recalcitrant organic compounds. The mechanism is achieved by the acceleration of a photoreaction in the presence of a catalyst [83]. Several catalysts such as In_2_O_3_, Fe_2_O_3_, TiO_2_, ZnO, CdS, and Ga_2_O_3_ can work as photocatalysts. At the present moment, titanium dioxide (TiO_2_) has been the compound that is widely investigated due to its ability to degrade organic pollutants and achieve satisfactory mineralization at a relatively low cost when compared to other destructive technologies [84].

**Figure 4:**
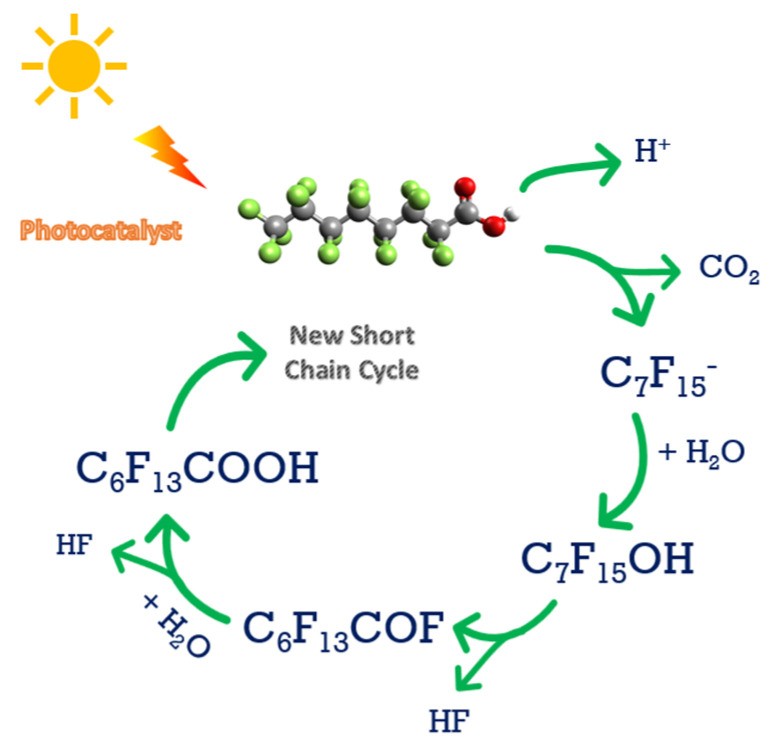
Photocatalytic method for PFAS degradation.

The photodegradation can be performed with a wide range of wavelengths, which overcomes the problem that it is difficult to destroy the C-F bond by direct photolysis [82,85,86]. However, after the photocatalyst absorbs light energy, it can generate negatively charged electrons and positively charged hole pairs, which will move onto the surface of the photocatalyst and react with the adsorbed PFAS [87]. Figure 4 shows the PFAS destruction using photocatalysis, which is a staged or sequential reaction. As can be observed in Figure 4, the first cycle eliminates one carbon and two fluorine atoms in the PFAS molecule, and subsequent cycles will further reduce the PFAS chain length until it fully degrades the PFAS molecule. Identified ways to enhance the photocatalysis efficiency and cost by adding carbon materials enhanced the range of the frequency of light absorbed by photocatalysts, with the possibility of applying sunlight as a sustainable energy source [87,88]. Furthermore, modifications to these semiconductor-based chemical compositions of photocatalysts, their morphology, and their size can significantly improve PFAA removal and mineralization [15]. In addition, the photocatalytic and hydrophilic properties of TiO_2_ make it close to an ideal catalyst due to its high reactivity, reduced toxicity, chemical stability, and lower costs [89].

Advantages: The photocatalytic treatment is a promising technology that can be performed at ambient temperatures and has a lower energy requirement than other thermal technologies [90]. Moreover, in contrast to many other AOP/ARP technologies, photocatalysis can be considered a highly sustainable process due to its low energy consumption and the possibility of consuming green energy. Additionally, the photocatalyst can be recycled [87,91].

Disadvantages: The degradation efficiencies of photocatalysis, when compared with other PFAS mineralization techniques, are relatively low, generating toxic intermediate reaction products. Additionally, it is more effective for PFOA and has minimal effects on PFOS [15]. The use of TiO_2_ for the PFOS is significantly ineffective as it only operates in the UV range and is hence unfavorable for PFOS adsorption and results in a significantly low degradation efficiency [84]. Besides its difficulty in degrading PFOSs, TiO_2_ is extremally challenging to recover from the treated solution, and it would require extra steps and further treatment to address this issue [15,92]. In addition, photocatalysis is impacted by the presence of co-contaminants, which can significantly lower the degradation efficiency [15,93].

### Field Applications

Research on the degradation of PFASs through photocatalysis is still in the laboratory, lacking a representative field application [82,84,86]. The degradation experiments are all done with deionized water [87]. Due to the fact this technology is at the developmental stage, it was not possible to perform an in-depth analysis. Therefore, this technology is listed in Table 1 to enable the reader to compare this technology with other more mature technologies.

## 5. Sonolysis

**Figure 5:**
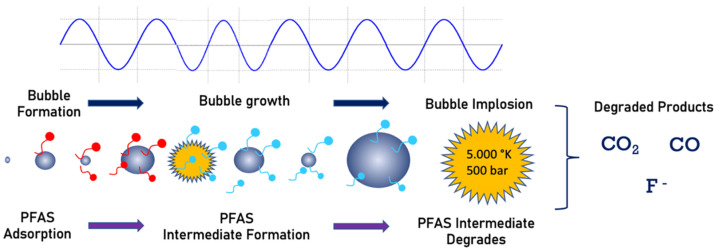
Sonolysis process of PFAS mineralization.

Sonochemistry uses an acoustic field to create chemical reactions in a solution. The mechanism consists of the generation and implosion of vapor bubbles. Organic pollutant degradation is achieved by a combination of radical reaction and combustion by pyrolysis [94,95,96]. The bubble collapse is the driving mechanism responsible for pyrolysis and combustion of organic compounds in the vicinity of the imploding bubble. The imploding bubbles produce very high temperatures (average 5000 K) [97,98]. During pyrolysis, due to extreme heat, water vapor will also be converted to H^−^ and OH^−^ radicals. These radicals react and degrade organic contaminants [99]. This technology has been widely used to degrade organic compounds, including PFCs, where the decomposition occurs at the bubble/water interface due to pyrolysis [4,13,100]. Figure 5 shows a schematic view of the PFAS destruction with sonolysis. The PFAS molecules adsorbed into the bubble surface will be pyrolyzed to several products during bubble implosion, as shown in Figure 5.

Research on sonochemical parameters indicates that ultrasonic frequency is highly important for the effective degradation of PFASs [96,101,102,103]. Although chemical activity can be further enhanced using a combination of frequencies [104,105], the production of cavitational bubble volume fractions is higher for dual-frequency than single-frequency reactors [105,106,107]. Along with frequency, power is also an important parameter of sonochemical degradation. Increasing the power increases the number of collapsed cavities formed, the maximum collapse temperature, and the sonochemical activity [108,109,110].

Advantages: Sonolysis has proven to be extremely effective in degrading PFOS and PFOA, where the reaction is mostly believed to be related to the pyrolytic reactions at the bubble/water interface [102,103,111]. As mentioned above, pyrolysis will induce localized incineration for a very short period of time of several ns. Interestingly, it has been observed that sonolysis can also degrade PFASs in soil with little residue remaining in the liquid phase [4,96]. Experiments with landfill leachate showed that co-contaminants did not impact the sonolysis efficiency of the degradation of PFOA and PFOS [108]. Furthermore, sonochemistry treatment can achieve complete defluorination without pretreatment and without the need for the addition of chemicals/additives and with higher kinetic reaction rates [105,112]. The technology does not need adjustments of temperature or pressure to work and has a low environmental impact [95,96].

Disadvantages: Sonolysis technology has been studied under controlled lab settings, which is not representative of field concentrations. Therefore, additional studies and experiments are needed in order to make it a fully mature technology. Research is still required to evaluate the cost associated with its implementation and maintenance. Energy consumption is a decisive factor that needs further investigation. Besides energy consumption and cost, several other factors require further attention, such as power density and the size of the transducer, the frequency and geometry of the reaction, as well as the physicochemical properties of environmental matrices [102]. These additional studies will be fundamental for scaling up this technology.

### Field Applications

Bench-scale: Investigation of high-frequency ultrasound’s (700 kHz–1040 kHz) use for the mineralization of seven individual PFAS compounds and a mixture of 24 standard PFASs, water with diluted AFFF, and concentrated investigation-derived waste (IDW) was tested, aiming to evaluate the interference of the matrix and PFAS constitution on destruction effectiveness [113]. The contaminated samples were treated in a 2 L reactor for 4 h in an atmosphere containing argon to enhance reaction kinetics. Results from a comparison of PFAS-contaminated groundwater and PFAS-spiked deionized water showed similar pseudo-first-order kinetics, presenting PFAS mineralization higher than 98% for the majority of PFASs within 2 h of treatment [113]. This study also showed the removal kinetics of a complex PFAS mixture (24 Mix) of short-chain PFASs in groundwater and in deionized water. The long-chain PFAS deionized water showed the highest destruction [113].

Large-scale: Gole et al. [105] investigated a large-scale multi-transducer sonochemical reactor with a capacity of 91 L and a dual-frequency approach to treat PFOS. The reactor had three 500 kHz transducers and nine 1 MHz transducers working simultaneously connected to six generators. This experiment evaluated three PFOS concentrations (0.32 mM, 2.6 mM, and 5.3 mM), aiming to analyze the efficiency of the sonochemical system. Results of fluoride and sulfate ions released in conjunction with the removal of the TOC test showed an optimum concentration of 2.6 mM. Despite the suggestion of treatment enhancement with a dual frequency system, they observed that single frequency was more efficient than dual frequency operation, with the increase in the fluoride ions concentration likely due to the lower intensity in the system [105].

Theoretical in-situ: To address the need for in-situ contaminated groundwater treatment, a theoretical system of ultrasonic reactors applied in a horizontal well, assuming two sonolytic reactors of 1.5 ft in length each, was presented with more details in the reference [114]. In this feasibility study, four groundwater-contaminated site samples were investigated and used to determine treatment time for the baseline at 70 ppt for each PFAS present. Although this treatment proved to be hypothetically possible, the variability of PFAS compounds and concentrations from different sites directly affected the treatment time and number of reactors necessary to achieve the concentration established, complicating its design and system feasibility.

## 6. Supercritical Water Oxidation (SCWO)

Supercritical water oxidation (SCWO) is an oxidation treatment process where OM is transformed into water, carbon dioxide, and a few other products depending on the wasted stream being treated. The process can treat an extensive range of wet wastes without dewatering. The SCWO technology has already proven to be effective in the destruction of toxic and persistent organic contaminants such PFASs [115].

**Figure 6:**
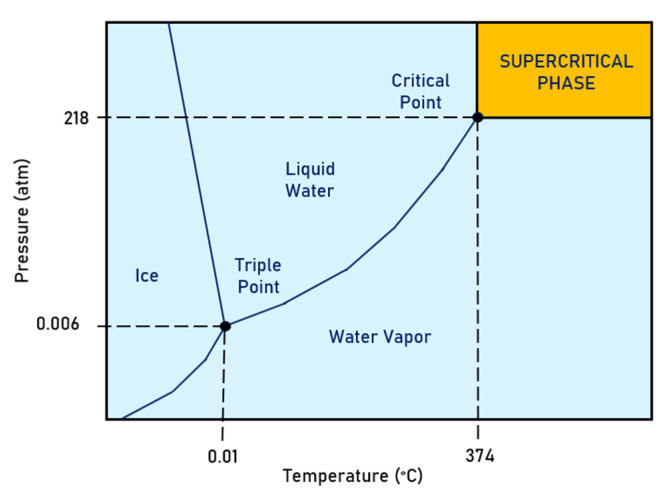
Water phase diagram highlighting the supercritical water region.

A supercritical water solution is a substance kept at a specific temperature and pressure above its critical point. Water above 374 °C and 218 atm becomes supercritical, a state where organic solubility significantly increases, and oxidation is enhanced [116,117]. Figure 6 shows the region where the water is in the supercritical state. It can be observed that, at the supercritical state, the liquid and vapor phases share similar properties. The fluid is neither a liquid nor a gas at this state and has properties of both states. Under these conditions, the fluid molecules start to behave differently. Supercritical water is highly expandable and compressible, and the mass transfer is unrestricted without distinct liquid and gas phases, promoting chemical reactions. Supercritical oxidation can break down compounds, such as PFASs, that do not oxidize at standard temperatures and pressures [116].

Advantages: SCWO is a destructive treatment where the compounds treated are degraded to simple elements. The absence of reaction byproducts, incompletely oxidized contaminants, or unreacted harmful oxidants is a distinctive characteristic of SCWO. The SCWO, when compared to other destructive technologies, is relatively faster, allowing it to reduce the size of the reactors and treat larger volumes at a low cost. The highly oxidizing environment helps the technology to not be impacted by the presence of co-contaminants and indeed allows the treatment of all types of organic contaminants with high efficiencies [117]. The SCWO can also be seen as an environmentally friendly technology when compared to other destructive approaches. The moderate temperatures (380–600 °C) prevent the formation of NOx and SOx compounds, which are highly toxic.

Disadvantages: The operational volume plays an important role in PFASs’ treatment with SCWO. Currently, it is not economically viable to work with volumes larger than 50,000 gallons/day of dilute waste streams. The cost of heating large masses of liquid inhibits the scaling up of this technology. Not only proving heat for a large volume can be a problem, but the mass transfer will also be an issue, as a large volume of liquid has to be pumped, and it will add an extra cost which may prevent the technology from being economically possible. Additionally, this technology does not comport waste streams that contain excessive grit or abrasive materials and soils [115]. During oxidation, SCWO produces many acidic species (H_2_SO_4_, HCl). This results in pH reduction, which can cause corrosion of the reactor if not addressed. An additional challenge of the SCWO is the precipitation of salts [118].

### Field Applications

Krause et al. performed a study with three organizations to treat/destroy PFAS in AFFF samples [117]. Battelle, Aquarden Technologies, and 374 Water were the selected organizations. In the Aquarden experiment, the reactor temperature was maintained at 590 °C, and the reactor operation pressure was 24 MPa [117]. A single test was conducted with influent and effluent samples analyzed for 12 compounds. The pH decreased over the entire experiment from 12.75 to 3.26 despite the alkaline buffer, indicating acid formation. Overall degradation efficiency was above 99% [117]. Table 1 shows the characteristics of each demonstration in detail. Although all three organizations were able to destroy a large percentage of PFAS, the total PFAS concentrations of the effluents were higher than the USEPA drinking water health advisory limit (PFOA and PFOS < 70 parts per trillion) [117,119].

## 7. Thermal Degradation/Incineration

**Figure 7:**
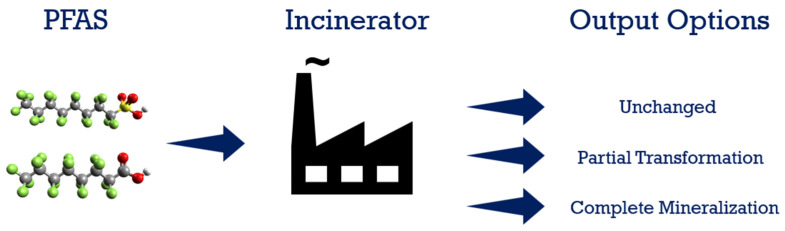
PFAS incineration.

Heat is applied in thermal treatment to treat and decompose/destroy materials. This is a complicated process, and the mechanism depends on many factors, including the operating conditions such as temperature, the environment of the heating chamber, gases present, residence time, mixture composition of waste streams, and chemical characteristics of materials to be treated. As for PFAS incineration, under ideal conditions (i.e., mineralization), the destruction of PFASs results in final products such as carbon monoxide, carbon dioxide, water, hydrogen fluoride, and sulfur molecules or sulphuric acid in the case of sulfur-containing PFASs. Due to their unique structure, PFAS are considered chemically and thermally stable, which translates into a requirement to apply higher temperatures and long residence time to achieve a satisfactory level of destruction. Figure 7 shows the PFAS destruction path through incineration. Incineration is considered to be a thermal oxidation treatment method, and it is carried out in an oxygen-rich atmosphere at high temperatures. Most hazardous waste incinerators operate from 980 °C to 1200 °C [120]. Since the thermal destruction of most organic compounds occurs between 590 °C and 650 °C, the expectation is that nearly total destruction of the organics in the waste will be achieved, including PFAS [120].

In 2020, the U.S. EPA [121] published a technical brief on the incineration of PFAS with the main conclusion that the effectiveness of incineration in destroying PFAS and their fate in terms of potential mixed fluorinated organic byproduct formation is not clearly understood. A significant concern is that incomplete destruction of PFAS can result in the formation of PIC (products of incomplete combustion), e.g., smaller PFAS molecules, which could be a potential hazard. Only a few studies are available related to PFAS incineration in full-scale operating facilities [41,122,123,124]. According to Solo-Gabriele et al., increasing incinerator temperatures decreased the total treated PFAS concentrations. However, not all PFAS species decreased with increasing temperatures [122]. There is an alarming report of higher concentrations of PFOA found in the air at the incinerator sites compared to upwind sites [123]. Public concern is that the incineration may spread PFAS and not break them down. This publication claims that the preliminary data show that soil and surface water near a commercial facility in Cohoes, New York, that has burned firefighting foam containing PFAS are contaminated with PFAS [125].

PFAS incineration can occur directly for PFAS-based materials, such as firefighting foams or indirectly via the incineration of waste containing PFAS, such as textiles, etc. [126]. Recently the Defense Department issued a ban on incinerating PFAS-laden items, with particular emphasis on the aqueous film-forming foam often used in training and combat situations [127]. In addition, under the 2022 National Defense Authorization Act [128], the military is now prohibited from incinerating PFAS-containing materials in accordance with the Clean Air Act [129]. Most incineration studies monitored a limited number of compounds, leaving the question of “unmonitored” PFAS unanswered [130]. Even though multiple studies were done on the thermal degradation of PFAS [131,132,133,134,135], only limited data [136,137] is available on directly detecting degradation products during field-scale incineration. The main obstacle is still the lack of both suitable emission sampling methods (including industrial field sampling) to capture PFAS compounds and analytical methods to identify/detect PFAS and their thermal decomposition byproducts. The question remains unanswered as to how significant is the portion of volatile species that escape the analysis.

Advantages: Thermal degradation/incineration is the widely available approach for managing contaminated solids, liquids, or gases using already built incinerator facilities [138], and many incineration facilities are already knowingly or unknowingly treating PFAS (e.g., consumer products, activated carbon regeneration). As previously mentioned, incineration facilities have already been deployed and are well-established in the industry. Therefore, the initial cost of implementing the technology will be significantly reduced compared to other PFAS-destructive technologies. Together, these advantages place them as a critical solution for managing PFAS-containing wastes [139,140].

Disadvantages: In general, waste byproducts in any incineration include bottom ash, which contains non-combusted products, and gas, containing tiny particles and volatile products [139]. According to Wang et al. [139] regarding PFAS incineration, the resulting ash and gas are both problematic. Ash contains inorganic fluorine and remaining PFAS bound to inorganic compounds such as calcium. Note that ash is typically sent to a landfill or repurposed. Particulates in the gas can be captured with electrostatic precipitators. However, HF is anticipated to be the main product of PFAS thermal conversion during incineration and is a corrosive/acidic gas. Capturing or removing volatile fluoride-containing byproducts might be an issue.

Any untreated PFAS or byproducts from incineration are released directly into the environment [139]. Therefore, the potential risk of secondary air and soil pollution and the return of PFAS into the environment is very high. In addition, incomplete destruction during thermal treatment/incineration could generate an unknown array of byproducts, which might be environmentally problematic. Since current knowledge of the fate of PFAS is limited, there is concern that PFAS incineration can release toxic gases (tetrafluoromethane, hexafluoroethane, fluoro-dioxins, fluoro-benzofurans, and perfluorinated carboxylic acids) [141,142].

### Field Applications

Most studies performed for PFAS destruction under thermal degradation/incineration have been performed using existing incineration facilities. The conditions do not allow for an accurate assessment of the efficiency of this technology, as discussed above. Therefore, this document does not report any field application of PFAS incineration.

## 8. Technology Comparison

Table 1 shows the experimental characteristics and PFAS source for each technology discussed in this review article. It also lists the reported degradation rates and the required energy per unit mass of PFASs. Since the incineration of PFASs is no more suggested as an alternative for disposal after the prohibition released by the U.S. Defense Department [128,129], it is not included in this comparison. Table 2 summarizes the advantages and disadvantages of each technology discussed here. It can be inferred from Table 2 that each technology presents its benefits and drawbacks.

Based on this analysis, the electrochemical oxidation and plasma reactor were tested in a pilot-scale field setting [44,72], appearing to have the most progress towards scaling up for real-world applications, its effective destruction range of 70–99%, as shown in Table 1. However, it is still far from being a reliable solution for managing PFAS-contaminated water, considering its volume, the creation of byproducts, and the cost of treatment. Applying electrochemical oxidation in synthetic samples, water impacted by aqueous film-forming foams and SBs exhibited relatively divergent treatment times. Samples sourced from naturally contaminated means (groundwater and wastewater) needed longer treatment time than PFAS-spiked water. The complexity of contamination with a wide range of polyfluorinated compounds created competition with intermediates. Even longer times were observed for SBs treatment, caused by its extremely high concentration compared to the other aqueous solutions.

A combined treatment approach between IXR and EO to deal with AFFF groundwater enhanced energy consumption compared to its stand-alone EO while achieving high destruction levels of PFOA and PFOS [44]. A more efficient mass transfer could explain the high contamination concentration results. The direct electron transfer from PFAS occurred more effectively on the anode surface, resulting in less electric energy wasted on concurrent water electrolysis [44,58,143]. Regardless of the incredible rates of defluorination and removal of PFOA and PFOS in most EO papers reviewed here (70–99%), the most significant concerns about this technique are the possible footprint from the release of its heavy metal electrodes and the production of harmful byproducts encountered in its treated water [42,45,63,64,65,66,68].

Plasma reactors demonstrated a quicker treatment time compared to electrochemical oxidation and sonochemistry. Although the mineralization of short-chain PFAS is the reason for the extension of this process, the slower removal of short-chain PFAAs is augmented by the fact that short-chain PFAAs can be generated as byproducts of degradation of long-chain PFAAs and/or precursors [79,144]. A high percentage of mineralization of PFOA and PFOS was observed for a plasma reactor treating AFFF-contaminated groundwater and ion exchange SB, showing higher removal efficiency in treating complex recalcitrant compounds [49,79].

Overall, the energy consumption of plasma reactors can be classified as median, even though it can diverge based on the style of plasma and reactor type. Unlike electrochemical oxidation, the treatment train of IXR and plasma reactor did not minimize energy consumption. The higher PFAS concentration and ionic strength encountered in SB solutions resulted in lower plasma area compared to the treatment of contaminated groundwater with a plasma reactor alone [79]. The chemical degradation pathway, experiment parameters, and acidification of treated water in plasma treatment still need further research. The formation of toxic byproducts such as chlorite and chlorate was only evaluated in a few studies [79,145].

Alternatively, sonochemical degradation is a promising technology proven by no formation of toxic derivatives after testing with actual contaminated water [113]. Its technology promotes the mineralization of PFAS molecules by cavitation bubbles generated for ultrasonic waves; conditions also occur with plasma technology. The PFAS removal in sonochemistry occurs simultaneously for long to short chains and from PFCAs to PFSAs; hence sonolysis is not considered to be chain-length selective [111,146,147]. In addition, high degradation efficiency is achieved in a relatively short treatment time. However, the energy consumption is higher than the other technologies considered here.

**Table 1:** Comparison of published PFAS destruction technologies investigation on water media.

	PFAS Source			Experimental Conditions									
Technology	AFFF	Still Bottom	Synthetic	PFASType	C_i_ (µg/L)	C_f_ (µg/L)	T (h)	Vol (L)	DE (%)	Energy Consumed (kWh/m^3^)	Mass of PFAS Destroyed (kg)	Energy per Mass (kWh/kg)	Ref.
Electrochemical Oxidation				Multi	1652	4.2	10	2	99.7412	256	3.2 × 10^−6^	1.6 × 10^5^	[35]
				PFHxA	870,000	87,000	1.5	1	90.0000	15.2	7.8 × 10^−4^	1.9 × 10^1^	[52]
			x	Multi	451	50.6	8	0.3	88.7804	99	1.0 × 10^−7^	2.5 × 10^5^	[45]
	x			Multi	539	126.4	8	0.3	76.5491	136	1.0 × 10^−7^	3.3 × 10^5^	[45]
		x		PFOA	3050	897	80	0.56	70.5902	0.161	1.21 × 10^−6^	3.98 × 10⁷	[44]
		x		PFOA	4080	609	80	0.415	85.0735	0.131	1.44 × 10^−6^	3.33 × 10⁷	[44]
		x		PFOS	4420	538	80	0.56	87.8281	0.094	2.17 × 10^−6^	2.21 × 10⁷	[44]
		x		PFOS	15,200	361	80	0.415	97.6250	0.071	6.16 × 10^−6^	7.79 × 10⁶	[44]
		x		8:2 FTS	193	50	120	0.56	74.0933	0.146	8.01 × 10^−8^	5.99 × 10⁸	[44]
		x		8:2 FTS	753	125	120	0.415	83.3997	0.148	2.61 × 10^−7^	1.84 × 10⁸	[44]
Sonochemical Degradation			x	PFOS	5001	0	3	0.6		208	3.0 × 10^−6^	4.2 × 10^4^	[103]
			x	PFOA	4141	0	2	0.6		208	2.5 × 10^−6^	5.0 × 10^4^	[103]
			x	PFOS	10,000	7200.0	1	0.1	28.0000	3333	1.7 × 10^−7^	1.2 × 10^6^	[100]
			x	PFOA	10,000	3700.0	1	0.1	63.0000	3333	3.8 × 10^−7^	5.3 × 10^5^	[100]
			x	PFOS	100	5	2.3	0.6	95.0000	448	5.7 × 10^−8^	4.7 × 10^6^	[148]
			x	PFOA	100	28	2.3	0.6	72.0000	1050	4.3 × 10^−8^	1.5 × 10^7^	[148]
			x	PFOS	9420	96	4		99.9898				[102]
			x	PFOS	9420	133	4		99.9858				[102]
			x	PFOS	9420	177	4		99.9812				[102]
			x	PFOA	140	1.2	4	2	99.1428				[113]
			x	PFOS	2.9	0.06	4	2	97.9310				[113]
			x	PFBS	10.5	1.06	4	2	89.9047				[113]
Plasma Reactor	x			PFOA	7.924	0.012	0.67	4	99.8486				[72]
	x			PFOS	2.264	0.012	0.67	4	99.4700				[72]
	x			PFPeA	10.452	2.432	1	4	76.7317				[72]
	x			PFBA	3.684	2.253	1	4	38.8436				[72]
		x		Mulit	261,067	0.82	6	0.75	99.9997	830			[79]
		x		PFOA	99,600	<0.015	2		>99	380–830			[79]
		x		PFOS	70,900	<0.035	2		>99	380–830			[79]
		x		Mulit	<1	BDL *	1.5		>99	1570–2370			[79]
		x		PFOA	1.5	<0.07	0.03		>99	380–830			[79]
		x		PFOS	1.75	<0.07	0.03		>99	380–830			[79]
		x		PFBA	6	0.26	1.5		95.6667	1570–2370			[79]
Supercritical Water Oxidation	x			Multi	12,176	0	2	1085.4	>99	1398	1.3 × 10^−2^	1.1 × 10^5^	[149]
	x			Multi	14,155	0	2	997.4	>99	1506	1.4 × 10^−2^	1.0 × 10^5^	[149]
	x			PFOA	26,200	240			99.0839				[117] a
	x			PFOS	930	0.14			99.9849				[117] a
	x			PFOS	30,599	0.33			99.9989				[117] b
	x			PFOS	30,251	0.29			99.999				[117] b
	x			Multi	40,454	8.64			99.9786				[117] b
	x			PFOS	190,000	8.57			99.9954				[117] c
	x			Multi	243,000	9.63			99.9960				[117] c
			x	PFOS	28,630		1		70.0000				[150]
Photocatalysis			x	PFOA	1000	690	1		31.000				[82]
			x	PFOS	1000	670	1		33.000				[82]
			x	PFNA	1000	0.26	2		99.974				[82]
			x	PFOA	0.5	0.27	2		46.000				[82]
			x	PFOS	0.5	0.06	2		88.000				[82]
			x	PFNA	0.5	0.05	2		90.000				[82]
			x	PFOS	100		4		75.000				[84]
			x	PFOA	100		4		90.000				[86]

Krause et al., 2022 [117] (a—Aquarden; b—Battelle; c—374 Water). * BDL = Base Detection Level.

**Table 2:** Summary of the main advantages and disadvantages of the PFAS destructive technologies.

TECHNOLOGY	ADVANTAGES	DISADVANTAGES
ELECTROCHEMICAL OXIDATION	Effective for long-chain PFASs. Efficient for highly concentrated PFASs. Effective for low-volume PFASs. Low environmental impact. Does not require pretreatment.	Widescale application. Inefficient for short-chain PFASs. Electrodes are expensive. Reduced electrode lifetime. High energy consumption. Toxic by-products. Forms short-chain PFAS
PLASMA	Effective for long-chain PFASs. Effective for short-chain PFASs. Low energy consumption. No chemical additives are needed. Short treatment time. Effective for highly concentrated PFASs. Effective against Co-contaminants.	Affects water’s pH, making it acidic. Forms short-chain PFASs. Its mechanism is not well understood. Longer time for short-chain treatment. The addition of chemicals is required. Nontargeted reactions can result in longer treatment time
PHOTOCATALYSIS	Low energy consumption. Performed at ambient temperatures. Sustainable technology. It can be recycled.	Low degradation efficiency. Inefficient for sulfonic groups. Toxic intermediate products. Additional treatment is needed. Affected by co-contaminants.
SONOLYSIS	Effective for long-chain PFASs. Effective for short-chain PFASs. Effective in soils and liquids. Effective for highly concentrated PFASs. Effective against co-contaminants. No chemical additives are needed. Does not require pretreatment. Efficient for highly concentrated PFASs.	Widescale application. High energy consumption. Its mechanism is not well understood. Optimization of ultrasonic and geometric parameters are needed to scaling up of technology
SUPERCRITICAL WATER OXIDATION	Effective for long-chain PFASs. Effective for short-chain PFASs Low environmental impact. Relatively quick treatment time	Not economically viable for large volumes. Affects water’s pH, making it acidic. Corrosion of the reactor. Precipitation of salts. Toxic intermediate products.
THERMAL DEGRADATION/ INCINERATION	Widescale application. Reduced capital cost. Effective for long-chain PFASs.	Toxic intermediate and final products. High environmental impact. Air and soil contamination. Toxic emission. Toxic by-products.

Analyzing experimental conditions of supercritical water oxidation, when compared with other techniques, demonstrates a high removal rate of PFOA and PFOS using AFFF samples; however, more in-depth analysis of parameters such as treatment time and an assessment study of possible products of its reaction is needed.

Looking only at energy consumption, photocatalysis stands out for its low energy requirement and the possibility of being connected to solar panels. However, the destruction of PFAS rates encountered in this review were significantly lower than the other technologies mentioned. Compared with sonolysis, its reaction times vary from several days to hours, requiring 2–30 times more energy per PFAS molecule degraded [102,146]. Besides this, the lack of experiments with complex samples, especially low PFOS removal, limits its ability to treat a complex mixture of PFASs.

Moreover, the identification of PFAS byproducts formation in photocatalysis from the breaking of long-chain PFASs to short-chain PFASs must be addressed since the short-chain is considered more persistent in the environment and difficult to remove with primary treatments. Consequently, more detailed research on methods for the degradation of PFASs is required to observe a clear comparison of techniques, investigating the efficacy of it in actual contaminated samples containing all the complexity of OM and different groups of PFAS contaminates.

## 9. Summary

This review compared the widely accepted as well as most recent emerging PFAS destruction/treatment technologies and their challenges. Even though all are potential candidates for implementation, many issues must be worked out to achieve a comprehensive technology to mineralize PFAS, considering efficiency and total cost of application. As observed in Table 2, every technology has its own advantages and disadvantages. There are many factors playing a significant role in deciding which methodology works better.

## 10. Conclusions

Some of the technologies considered in this review work better for long-chain PFAS in highly-concentered streams, while others work better in low-volume systems and are effective for short-chain PFAS. Some technologies are environmentally friendly, whereas others are toxic to the environment. The capital and maintenance costs also play an important role, as well as the energy consumption over the treatment period. These variables make it extremely difficult to favor one technology over another. The treatment site, available cost, efficiency target, PFAS initial concentration, and the treatment duration are decisive factors that will shape the decision-making for which technology will be preferable.

From the state-of-the-art presented in this document, electrochemical oxidation and plasma reactor appears to have considerable advance in scaling up a system for treating actual PFAS samples. However, sonochemical degradation is another promising technology with no formation of toxic derivatives with high degradation efficiency, but still in the laboratory scale of development.
